# Leaching Characteristics and Mechanisms of Fluorine and Phosphorus from Phosphogypsum

**DOI:** 10.3390/molecules30010005

**Published:** 2024-12-24

**Authors:** Wanqiang Dong, Xiangyi Deng, Liqi Chai, Yuefei Zhang, Haodong Chen, Hanjun Wu, Ru’an Chi

**Affiliations:** 1 School of Resources and Safety Engineering, Wuhan Institute of Technology, Wuhan 430205, Chinachailiqi1021@163.com (L.C.); 2 School of Chemistry and Enviromental Engineering, Wuhan Institute of Technology, Wuhan 430205, China; zyfwit@126.com (Y.Z.); wuhj1204@wit.edu.cn (H.W.); 3 Hubei Three Gorges Laboratory, Yichang 430073, China

**Keywords:** phosphogypsum, regional environmental risks, release behavior, Visual MINTEQ, mechanism

## Abstract

As a large-volume industrial solid waste generated during the production of wet-process phosphoric acid, the primary disposal method for phosphogypsum (PG) currently involves centralized stockpiling, which requires substantial land use. Additionally, PG contains impurities, such as phosphorus, fluorine, and alkali metals, that may pose potential pollution risks to the surrounding environment. However, the mechanisms governing the co-release of phosphorus and fluorine impurities alongside valuable metal cations during leaching remain unclear, posing challenges to efficient disposal and utilization. This study compares the leaching characteristics of cations and anions in PG of different particle sizes through static pH leaching experiments. Using Visual MINTEQ simulation combined with XRD, XPS, and FT-IR characterization methods, we analyzed the leaching mechanisms and key controlling factors for various metal elements and inorganic elements, like phosphorus and fluorine, under different pH conditions. The experimental results show that Ca, Al, Fe, Ti, Ba, Sr, Y, and PO_4_^3−^ in PG are more easily released under acidic conditions, while Si, Zn, Co, and F are primarily influenced by the content of soluble components. The dynamic “dissolution–crystallization” reaction of CaSO_4_·H_2_O significantly impacts the leaching of fluorine, and the XRD, XPS, and FT-IR characterization results confirm the presence of this reaction during the leaching process. This research provides theoretical guidance for the environmental risk assessment of stockpiled PG and the recovery of phosphorus, fluorine, and valuable metal resources from PG.

## 1. Introduction

Phosphogypsum (PG) is a major industrial solid waste generated during wet-process phosphoric acid production [1,2,3]. Globally, the annual production of PG is estimated to be approximately 300 million tons [4,5], making it one of the largest sources of newly generated industrial solid waste [6]. In contrast to countries, like Japan and Germany, where gypsum resources are limited and PG can be completely utilized [7], major producers of PG, such as China, Morocco, and the United States, encounter difficulties in the safe and resource-efficient disposal of PG. This challenge has become a significant constraint on the sustainable development of the wet-process phosphoric acid industry [8,9,10,11]. Due to the characteristics of phosphoric acid production using the wet process [12,13], a large number of impurities are transferred from the phosphate concentrate to the PG. In addition, some free phosphoric acid is transferred to PG during product separation, resulting in high levels of phosphorus, fluorine, alkali metals, and other impurities in PG [14,15,16,17]. When disposed of through harmless stockpiling, PG occupies significant land resources while posing potential pollution risks to the surrounding environment. Among the impurities in PG, the release of water-soluble phosphorus, fluorine, and heavy metal impurities is particularly noteworthy, as it presents significant environmental risks to the soils near the storage sites [18,19,20,21].

PG has mineralogical properties, so the growth of gypsum crystals in PG is similar to the mineralization process [22,23,24]. Because the sulfuric acid in the phosphoric acid reaction solution of the wet process is in a supersaturated state, and due to varying crystal growth conditions and times, the crystal size of PG products varies widely, ranging from 0 to 830 µm [25,26,27]. Previous investigations indicated that the distribution characteristics of phosphorus and fluorine impurities in PG are remarkably affected by the size of PG particles, and the distribution and occurrence states of these impurities directly influence their release behavior [28]. Zhao et al. [29] found that water-soluble phosphorus and fluorine in PG can be effectively leached, whereas water-soluble fluorine is more challenging to release. Research by Wu [30] showed that the concentration of toxic and harmful substances in PG leachate is higher than that of PG itself, and it has a greater tendency to diffuse and migrate. Notably, in solid–liquid leaching systems, the chemical behavior of valuable metal cations in solution noticeably affects the migration and transformation of anions [31,32]. When phosphorus and fluorine impurities are leached from PG into the leachate, the metal cations in the PG are often leached simultaneously and become toxic and harmful pollutants in the leachate [33,34,35,36,37]. Currently, most of the research on PG is focused on the release of phosphorus and fluorine, and limited studies are conducted on the mutual effects of phosphorus, fluorine, and valuable metal cations during impurity release [38,39,40,41,42]. Therefore, a thorough understanding of the occurrence characteristics and release behavior of phosphorus, fluorine, and valuable metal impurities in PG with various particle sizes could provide a methodical basis for the environmental risk assessment of PG and technical support for the effective recovery and utilization of phosphorus, fluorine, and valuable metal cations in PG.

This study meticulously investigates the acid–base neutralization capacity of PG samples by conducting a pH-static leaching experiment. Our goal is to thoroughly compare the leaching characteristics of cations and anions in PG with varying particle sizes, emphasizing the critical leaching behaviors of phosphorus and fluorine. By analyzing the leached quantities of cations and anions across different particle sizes under controlled static conditions, we unveil significant insights that are essential for understanding the environmental implications and potential applications of PG. Visual MINTEQ 3.1 geochemical software is utilized to simulate the changes in leachate species in the presence of various pH conditions. Saturation indices (SI values) are then appropriately evaluated for the main potential species, and the leaching mechanisms and main control mechanisms of water-soluble phosphorus and fluorine impurities in PG are suitably analyzed via characterization-based methodologies, including XRD, FTIR, and XPS. This investigation aims to establish a fairly solid theoretical basis for the environmental risk assessment and control pathways of phosphorus and fluorine release from PG.

## 2. Results and Discussion

### 2.1. Sample Analysis

#### 2.1.1. Particle Size Distribution of PG

**Figure 1:**
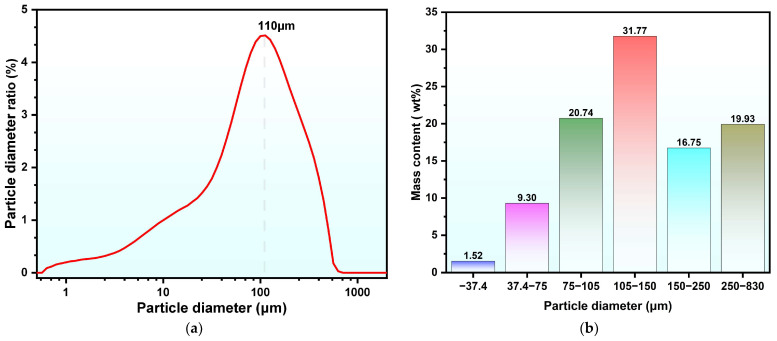
Particle size analysis of the PG samples. (**a**) statistical particle size distribution; (**b**) sieve particle size distribution.

Studies have shown that the occurrence state of phosphorus and fluorine impurities in PG is significantly affected by particle size [23]. In particular, the content of water-soluble phosphorus is considerably higher in coarser PG particles than in finer ones. Based on this, the particle size distribution and chemical composition of PG samples of different particle sizes were determined, as shown in Figure 1a,b. PG particles were relatively coarse, with the largest proportion found in the 105–150 μm range, representing 31.77% of the total mass, consistent with the particle size analysis results.

#### 2.1.2. Chemical Composition of PG of Different Particle Sizes

**Figure 2:**
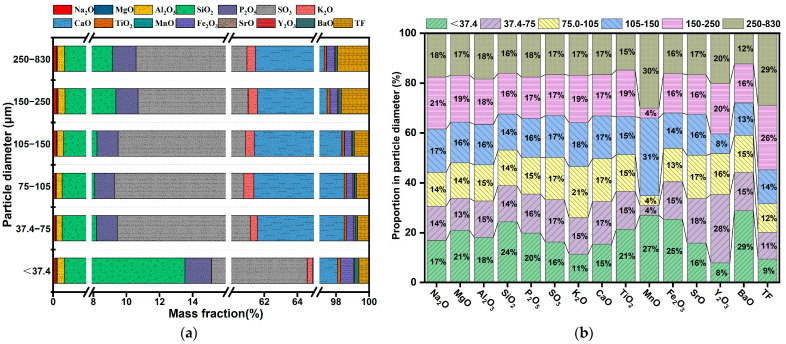
Chemical composition of PG at various particle sizes: (**a**) chemical element content at different particle sizes; (**b**) element distribution in different PG particle sizes.

To understand the enrichment characteristics of phosphorus, fluorine, and valuable metals in PG with various particle sizes, the chemical composition of PG in various particle size ranges was analyzed, with the results presented in Figure 2a. Figure 2a illustrates that PG particles smaller than 37.4 μm contain remarkably more quartz than other particle sizes. Further, fluorine impurities are more concentrated in the size range of 150–830 μm, whereas the valuable metals (Si, P, Fe, Mg) are more abundant in the size range of <37.4 μm. The distribution of these elements by particle size (see Figure 2b) indicates that Si, P, Fe, and Mg are mainly distributed in the finest particles (<37.4 μm), whereas fluorine is enriched in coarser particles (250–830 µm), with a notable enrichment effect seen in the coarser PG.

### 2.2. Phosphorus and Fluorine Release Characteristics in Static Leaching of PG

#### 2.2.1. Cation Release Characteristics

**Figure 3:**
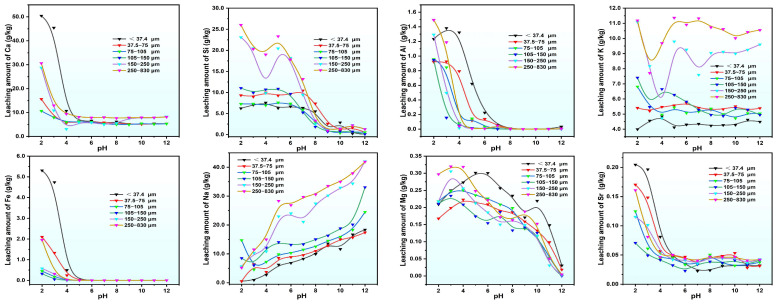

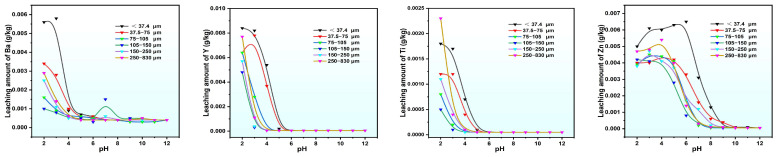
Leaching quantities of metal elements in PG leachate subjected to various pH conditions.

The variation in the quantity of metallic elements released during the static leaching of PG with various particle sizes has been presented in Figure 3. Some elements, such as Ca, Al, Fe, Ti, Ba, Sr, and Y, exhibit an “L-shaped” leaching curve, with higher release levels under acidic conditions and exhibit a descending with increasing pH. For pH levels higher than 4, the release levels of Fe, Ti, Ba, and Sr decrease significantly, and above pH = 6, these elements along with Y are almost completely retained, showing a constant trend in particle sizes. The elements Si, Zn, and Co exhibit an inverted “Z-shaped” leaching curve with maximum release at pH = 2 to 5, although the total leaching remains relatively stable over the pH range, indicating that their release is likely determined by the soluble fraction within PG. The Na content in leachate increases linearly with rising pH, primarily due to the addition of NaOH during pH adjustment. Mg and Ni display a linearly decreasing trend with pH, with the release of Mg and Ni being relatively unaffected by pH changes below pH = 9, indicating that the leaching capacity may approach the maximum limits constrained by their total content in the sample.

**Figure 4:**
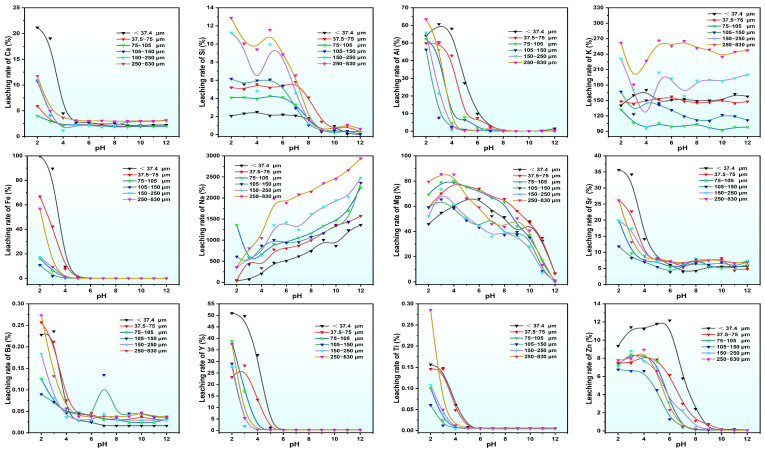
Leaching rate of metal elements in PG leachate under different pH conditions.

The leaching rates of various metals and silicon in PG with various particle sizes under different pH conditions are illustrated in Figure 4. The elements Ca, Al, Fe, Ti, Ba, Sr, and Y exhibit “L-shaped” leaching rate curves, with high rates under acidic conditions and decreasing rates as the pH increases. In the case of pH = 2, the leaching rate of Al in particles smaller than 37.4 µm reaches 50.4% and Fe reaches nearly 100%. The finer particles show significantly higher Fe leaching rates than the coarser ones, most likely due to better solid–liquid contact conditions in acidic solutions. The dissolution rates of Ti and Ba are below 0.5% across particle sizes, indicating good chemical stability for the mineral phases containing Ti and Ba in PG. The rate of calcium leaching shows a consistent pattern across particle sizes, with a dissolution rate of 21.16% for particles smaller than 37.4 μm under acidic conditions, which rapidly reduces with increasing particle size. Si and Zn inhibit the reverse “Z-shaped” leaching rate curve with the highest leaching rates at the range of pH = 2~5. Once the pH exceeds 6, Si release decreases sharply, with minimal leaching above pH = 8, probably due to the presence of Si as silicate minerals in the coarser PG and as quartz in the finer particles. Mg leaching rates lessen linearly with pH, with higher rates for particles below pH = 9 because Mg is primarily associated with chlorite, biotite, and rutile minerals that are uniformly distributed in particle sizes.

#### 2.2.2. Anion Release Characteristics

**Figure 5:**
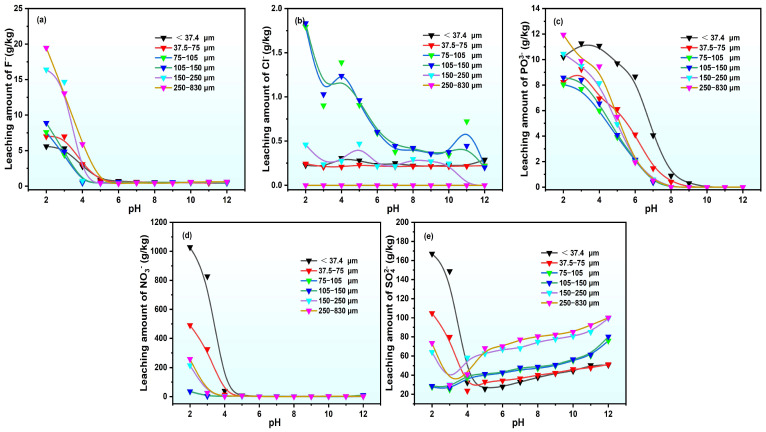
Leaching quantity of anions in PG leachate subjected to various pH conditions (**a**) F^−^, (**b**) Cl^−^, (**c**) PO4^3−^, (**d**) NO_3_^−^, and (**e**) SO_4_^2−^.

The release of anions during the static leaching of PG with different particle sizes is illustrated in Figure 5. The release of F^−^, PO_4_^3−^, and NO_3_^−^ rapidly lessens with growing pH, with F^−^ and NO_3_^−^ almost ceasing at high pH = 5. The Cl^−^ leaching remains continuously low, whereas PO_4_^3−^ release only substantially reduces at pH values above 8, and SO_4_^2−^ release initially increases and then decreases as pH rises, with a critical point around pH = 4.

**Figure 6:**
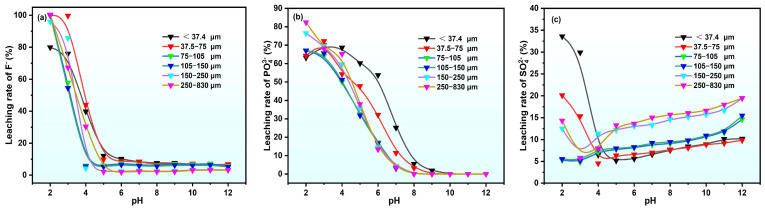
Anion leaching rates in PG static leachate in the presence of various pH conditions: (**a**) F^−^, (**b**) PO_4_^3−^, and (**c**) SO_4_^2−^.

As demonstrated in Figure 6, the leaching rates of anions in leachate exhibit dissimilar patterns. As the pH decreases below 5, the leaching rate of F^−^ increases rapidly, with minimal differences in F^−^ leaching rates across particle sizes at constant pH levels. In the case of pH = 5, the leaching rate of F^−^ for particles smaller than 37.4 μm reaches 12.12%, significantly higher than for coarser particles. This is probably attributed to the better solid–liquid contact and faster “dissolution–recrystallization” reactions of gypsum mineral phases, leading to a more complete release of F^−^ impurities.

**Figure 7:**
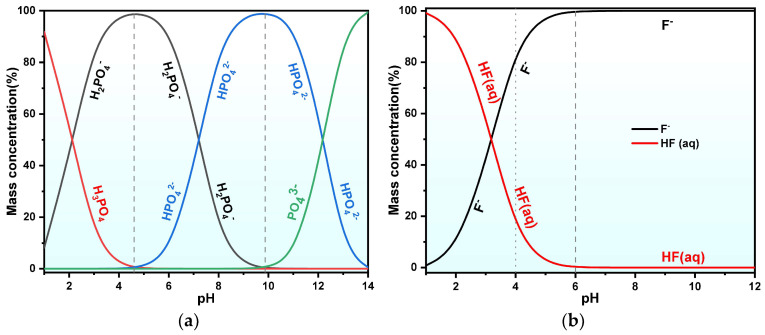
Speciation diagram of soluble phosphorus and fluorine subjected to various pH conditions. (**a**) pH phase diagram of water-soluble phosphorus; (**b**) pH phase diagram of water-soluble fluoride.

The pH-F phase diagram (Figure 7b) indicates that at pH = 5, F^−^ exists in a water-soluble form, with comparable Ca release across particle sizes, resulting in higher F^−^ in fine-particle leachate. The leaching rate of PO_4_^3−^ is less affected by the particle size, increasing rapidly as pH reduces below 8, with no significant difference in sizes at constant pH levels. In the case of pH > 8, the leaching rate of PO_4_^3−^ substantially lessens with further increases in pH, as illustrated in the pH-PO_4_^3−^ phase diagram in Figure 7a, where PO_4_^3−^ precipitates as Ca_5_(PO_4_)_3_OH at pH > 5. The leaching rate of SO_4_^2−^ is remarkably affected by particle size. Coarser PG has a relatively high gypsum content and crystallinity, releasing Ca^2+^ through the “dissolution–recrystallization” of gypsum mineral phases, and forming Ca_5_(PO_4_)_3_OH precipitate, and this in turn results in higher SO_4_^2−^ leaching rates in coarser PG.

### 2.3. Visual MINTEQ Simulation Calculations

#### 2.3.1. Species Changes During Leaching

**Figure 8:**
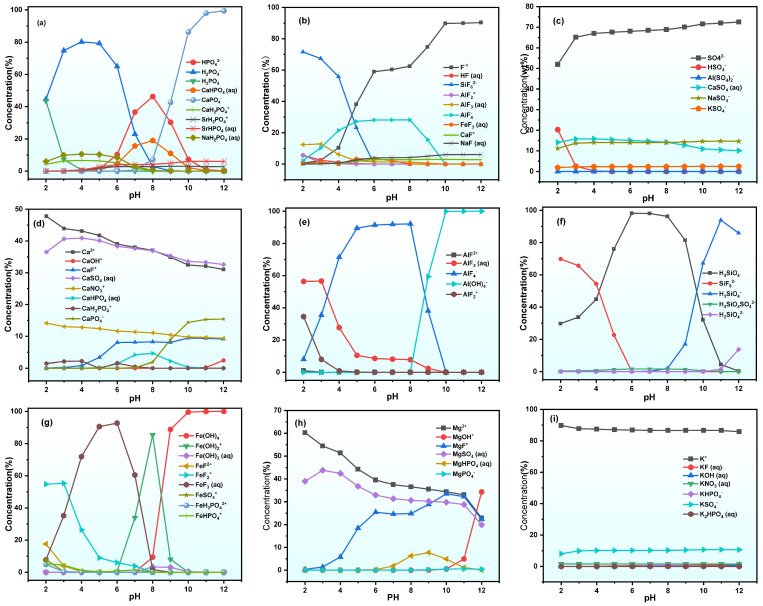
Simulation results of variations in species in the PG leachate. (**a**) P; (**b**) F; (**c**) SO_4_^2−^; (**d**) Ca; (**e**) Al; (**f**) Si; (**g**) Fe; (**h**) Mg; (**i**) K.

Using Visual MINTEQ software, elemental species variations for key elements in PG during static leaching were also simulated (see Figure 8). Figure 8a demonstrates that, within the pH range from 2 to 12, the PO_4_^3−^ species in PG leachate include H_3_PO_4_, H_2_PO_4_^−^, FeHPO_4_^−^, and CaPO_4_^−^. For the case of pH < 4, the concentration of water-soluble H_3_PO_4_ quickly reduces with increasing pH, while the concentration of H_2_PO_4_^−^ first increases significantly in the pH range of 2–6 and then decreases. As the pH rises to 8, the concentration of H_2_PO_4_^−^ sharply decreases to about 2% and a new species called CaPO_4_^−^ appears, whose concentration increases significantly with a further increase in pH. As the pH level increases from 8 to 10, the concentration of CaPO_4_^−^ species increases from 7.33% to 86.36%. What is more, CaHPO_4_ is present in the pH range of 5–10 but disappears above pH = 10.

Fluorine species exhibit a simpler distribution pattern. As illustrated in Figure 8b, the dominant fluorine species in the leachate include F^−^, SiF_6_^2−^, and AlF_4_^−^. In the pH range of 2–6, SiF_6_^2−^ is the primary fluorine species. However, with increasing pH, F^−^ and AlF_4_^−^ become the predominant fluorine species.

As depicted in Figure 8c,i, SO_4_^2−^ and potassium species display stable forms in leachate at the pH level. SO_4_^2−^ remains about 70% at all pH values, with calcium sulfate and potassium sulfate contributing about 10%. Potassium primarily exists as K^+^ (90%) and KSO_4_^−^ (10%), indicating that SO_4_^2−^ and K^+^ in PG do not significantly affect the “dissolution-precipitation” behavior of specific solution components.

Figure 8e,g illustrate that Fe and Al species undergo similar deformations. Regarding the pH range below 10, Fe and Al species mainly exist as metal fluorine ions. At pH = 8, metal hydroxides start to appear, and after pH = 10, metal hydroxides become the dominant species.

#### 2.3.2. Precipitation Mechanism Analysis

**Figure 9:**
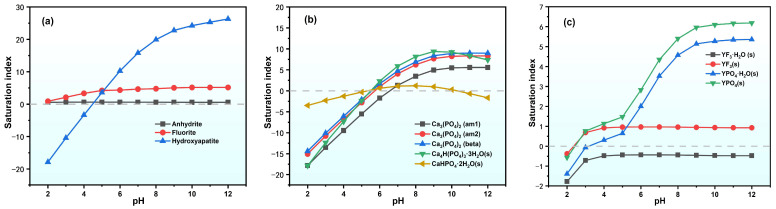
Variations in the SI species in the PG leachate. (**a**) typical fluoride species; (**b**) calcium phosphate species; (**c**) Yttrium metal salt species.

The precipitation mechanism of impurities, such as phosphorus and fluorine, in PG during the leaching process was also examined based on the saturation index (SI) calculations for the key mineral phases (Figure 9). The plotted results in Figure 9a,c reveal that under acidic conditions (pH < 5), the SI values for fluorite (CaF_2_) and rare-earth fluoride (YF_3_) remain at relatively low levels, indicating that fluorine ions exhibit high solubility and migration potential under acidic leaching conditions; however, when the pH exceeds 5, the SI values of CaF_2_ and YF_3_ increase significantly. This means that the precipitation rates of CaF_2_ and YF_3_ are probably the main controlling factors for the fluorine release rates from PG.

For phosphate species, Figure 9b,c illustrate that under highly acidic conditions (pH < 3), phosphate ions initially tend to react with Y^3^^+^, forming stable phosphate precipitates. With further increases in pH, PO_4_^3−^ ions gradually react with calcium ions and form different species of calcium phosphate. The dominant species observed are Ca_5_(PO_4_)_3_OH and Ca_3_(PO_4_)_2_, which exhibit high stability in leachate. This suggests that, for PG with low yttrium content, Ca_3_(PO_4_) is probably the main controlling factor for phosphate release.

### 2.4. Analysis of Phosphorus and Fluorine Release Mechanism in PG

#### 2.4.1. XRD Characterization

**Figure 10:**
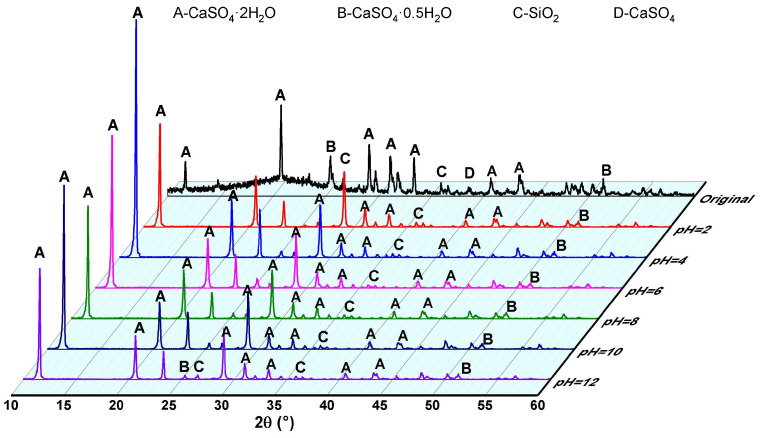
Contents of various phosphorus forms in PG before and after static leaching.

XRD spectra of PG samples before and after static leaching at pH levels of 2, 4, 6, 8, 10, and 12 are presented in Figure 10. The plotted results indicate that the diffraction peak at 11.7° is significantly enhanced post-leaching, while the intensity of the peak at 20.8° gradually reduces with increasing pH. In addition, a new diffraction peak at 23.3° appears in the leached samples. These diffraction peaks correspond to specific crystal planes in PG: (020), (121), (040), and (002) for peaks at 11.7°, 20.8°, 23.3°, and 29.2°, respectively [43,44]. The increase in intensity and width of the (020) plane diffraction peak in leached samples confirms that the “dissolution–recrystallization” of CaSO_4_·2H_2_O occurred during leaching. Furthermore, phosphate impurities are believed to replace sulfate ions (SO_4_^2−^) in the gypsum crystal structure, as HPO_4_^2−^ and soluble phosphorus exist as HPO_4_^2−^ in the pH range of 4–10. Therefore, as leaching proceeds at pH values above 4, an increase in phosphorus is coprecipitated, leading to the observed reduction in diffraction intensity at 11.7°. Notably, the characteristic diffraction peaks of hemihydrate gypsum (CaSO_4_·0.5H_2_O) at 25.4° and anhydrite at 38.6° are remarkably reduced, indicating that dissolution of these crystalline forms has occurred.

#### 2.4.2. FTIR Characterization

**Figure 11:**
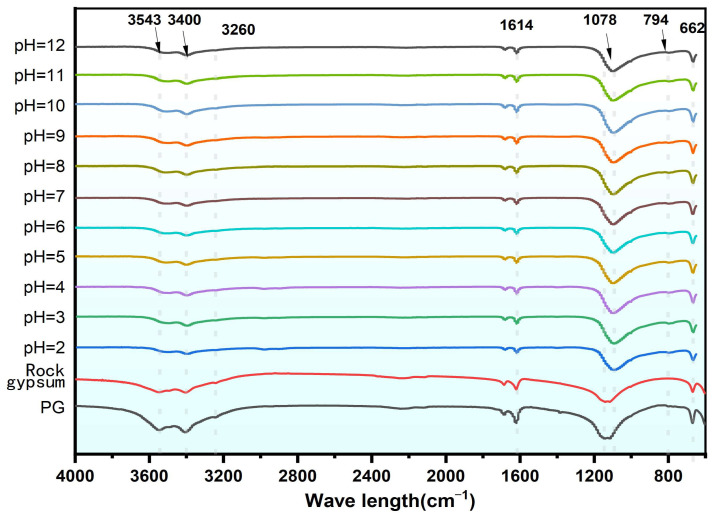
FTIR analysis spectra of various PG samples.

FTIR analysis was performed on PG samples before and after leaching under different pH conditions and also on natural gypsum samples. The plotted FTIR spectra (Figure 11) reveal that the intensity of the absorption peak of the symmetric stretching vibration of the O-S-O group at 662 cm^−1^ in the leached samples was lower than that of raw PG, which is more aligned with natural gypsum, suggesting a reduction in impurities and an increase in purity. In natural gypsum, the weak absorption peak at 794 cm^−1^, which is essentially attributed to the in-plane bending of HPO_4_^2−^, was absent, indicating that natural gypsum contains less coprecipitated phosphorus than detectable by FTIR. During leaching, a remarkable blue shift was observed in the antisymmetric stretching vibration from 1142 cm^−1^ to 1078 cm^−1^, indicating a decrease in the length of the S=O bond in SO_4_^2−^ [45,46]. This is indicative of a dissolution–recrystallization process that occurs in CaSO_4_·2H_2_O. Furthermore, a distinct reduction in the H_2_O stretching vibration peaks at 3400 cm^−1^ and 3543 cm^−1^ corroborates the “dissolution–recrystallization” process of CaSO_4_·2H_2_O during leaching.

#### 2.4.3. XPS Characterization

**Figure 12:**
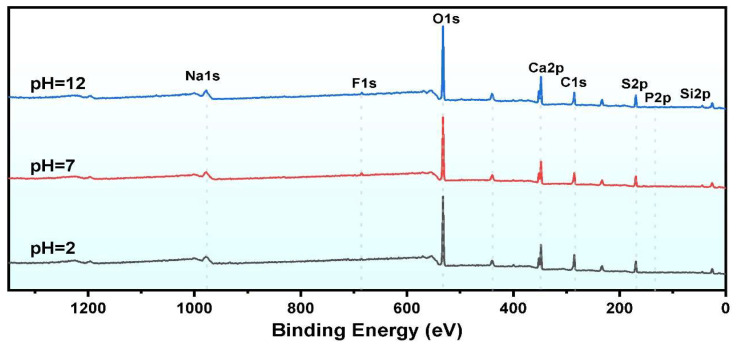
XPS survey spectra of leached samples under different pH conditions.

**Figure 13:**
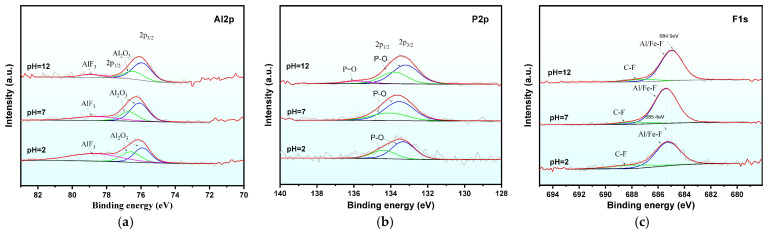
Peak fitting spectra of XPS for the static leaching samples: (**a**) Al 2p peak fitting spectrum, (**b**) P 2p peak fitting spectrum, and (**c**) F1s: peak fitting spectrum.

XPS was employed to analyze the chemical states and composition of elements on the surface of PG samples at pH = 2, 7, and 12. Figure 12 illustrates the survey spectra, which show significant changes in the binding energies of Al 2p, F 1s, and P 2p post-leaching. In particular, a strong binding energy peak at 685.21 eV was observed in the high-resolution F 1s spectrum in the pH 12 leached sample, while this peak was weaker in the pH 2 sample. Similarly, a P 2p binding energy peak was observed at 133.51 eV, corresponding to CaHPO_4_. The resolved high-resolution spectra of Al 2p, P2p, and F1s in Figure 13 reveal the formation of a new peak at 77.8 eV in the Al 2p spectrum, which is absent in raw PG, indicating the formation of new chemical bonds with F^−^. The strength of the peak at 77.8 eV lessened with increasing pH, indicating that high pH conditions prevent the formation of this bond. At pH 12, the P 2p spectrum exhibited a new peak at 135.9 eV attributed to the P=O bond in Ca_3_(PO_4_)_2_, indicating that phosphates may precipitate as Ca_3_(PO_4_)_2_ under alkaline conditions.

High-resolution XPS peak fitting results for the Al 2p, P 2p, and F 1s spectra are presented in Figure 13. In the Al 2p spectrum (Figure 13a), three peaks were observed after fitting, and the binding energy peak was observed at 77.8 eV, which was absent in the PG prototype, indicating the formation of a new chemical bond between F^−^ and Al during static leaching. As the pH of the leachate increased, the intensity of the 77.8 eV peak gradually decreased, indicating that high pH conditions are unfavorable for the formation of this compound.

In the P 2p spectrum (Figure 13b), the high-resolution spectra for the samples leached at pH 2 and 7 show two fairly coincident peaks at 133.2 eV and 134.5 eV, corresponding to the P 2p_3_/_2_ and P 2p_1_/_2_ states, associated with the P-O bond in CaHPO_4_. At pH = 12, the spectrum contains a third peak at 135.9 eV, which is attributed to the P=O bond in phosphate, based on the solubility–pH phase diagram for soluble phosphorus, where PO_4_^3−^ is the dominant form in the pH range of 10–12. According to the findings of KIM S. SIOW et al. [47,48], the binding energy of PO_4_^3−^ in phosphorus oxides is approximately 134.0 eV, which further confirms that the 135.9 eV peak in the P 2p spectrum corresponds to PO_4_^3−^ in Ca_3_(PO_4_)_2_.

For the F 1s spectrum (Figure 13c), two peaks were observed in the samples leached at pH = 2 and 7, located at 685.4 eV and 687.9 eV, corresponding to metal fluorine [49,50]. The high binding energy of 687.8 eV is associated with inorganic fluorides or AlF_3_. Based on the high-resolution peak fitting for Al 2p, the peak at 687.9 eV in the F1s spectrum can be attributed to the binding energy of AlF_3_.

## 3. Materials and Methods

### 3.1. Materials and Reagents

The PG samples utilized here were obtained from a phosphoric acid production facility in Yichang, Hubei, which produces PG as a byproduct of the “dehydration process” for wet-process phosphoric acid. To this end, the needed samples were collected via standard sampling methods, dried to a constant weight in an oven at 45 °C, and stored in a desiccator for further use. Analytical-grade HNO_3_ and NaOH, purchased from Sinopharm Group, were employed for pH adjustment. The cation concentration in the leachate was measured using an Agilent 7500s inductively coupled plasma–mass spectrometer (ICP-OES) (Agilent Technologies, Santa Clara, CA, USA), whereas the anion concentration was evaluated by a Metrohm IC930 ion chromatograph (IC) (Metrohm, Herisau, Switzerland). The mineralogical composition of PG was examined via an XRD-6000X X-ray diffraction (XRD) apparatus (Shimadzu, Kyoto, Japan), while the chemical compositions were investigated with an AXIOS X-ray fluorescence (XRF) spectrometer (PANalytical, Almelo, The Netherlands). X-ray photoelectron spectroscopy (XPS) analysis was performed using a Thermo Scientific K-Alpha XPS instrument (Thermo Fisher Scientific, Waltham, MA, USA).

### 3.2. Experimental Methods

Static leaching tests were performed following the static pH leaching approach specified in EN14997:2015 [51]. During leaching, an automatic potentiometric titrator was utilized to control the addition of NaOH and HNO_3_ to maintain the leaching system at pH values of 2, 3, 4, 5, 6, 7, 8, 9, 10, 11, and 12, respectively. After leaching, the leachate was centrifuged, and the supernatant was analyzed for metallic elements (Ca, Al, Fe, Ti, Ba, Sr, Y, Zn, Co, Na, Mg, and Ni), non-metallic elements (Si), and anions (SO_4_^2−^, NO_3_^−^, Cl^−^, PO_4_^3−^, and F^−^). Then, the remaining solid was dried, ground, and sieved to further characterize the samples using FTIR, XRD, and XPS. The XPS analysis was appropriately calibrated based on the C 1s spectrum at 284.80 eV, and data fitting was suitably performed using Avantage software 16.0 to analyze the elemental composition and chemical bonding forms on the PG surface. The leaching rate was calculated using the following formula:

$$R=\frac{\left(C_{t}-C_{0}\right)}{M}\times 100\%$$

where $C_{t}$ and $C_{0}$ represent the element concentrations at leaching time and the initial concentration, respectively, and *M* denotes the content of the corresponding element in the sample.

### 3.3. Visual MINTEQ Calculation

Based on the accurately measured concentrations of cations and anions in PG leachate under different pH conditions, the Visual MINTEQ software was utilized to simulate changes in key species during static leaching of PG with various particle sizes subjected to various pH conditions [52,53,54]. The simulation of leaching behavior classifies chemical substances in the aqueous solution into “components” (reactants in chemical reactions) and “species” (products formed after reactions). The variations in components and species are related through equilibrium constants, and an iterative algorithm is effectively implemented to simulate processes, such as complexation, dissolution–precipitation, and redox reactions, in a solution [55,56,57,58,59]. Since PG contains various cationic and anionic impurities, simulation calculations were adopted to explore the release mechanisms of phosphorus and fluorine impurities in PG and the “dissolution-precipitation” control mechanisms of the corresponding chemical products.

## 4. Conclusions

This study carried out an in-depth investigation of the synergistic release behavior and mechanisms of phosphorus and fluorine impurities along with valuable metal cations during the leaching process of PG. The main obtained results can be summarized as follows:

(1) In PG, some elements, such as Ca, Al, Fe, Ti, Ba, Sr, Y, and PO_4_^3−^, are easily leached under acidic conditions, and the level of leaching shows a descending trend with increasing pH. The leaching of dissolved Si, Zn, Co, and F^−^ is primarily determined by their soluble content in PG. The F^−^ release in <37.4 μm PG particles is significantly higher than in coarser particles, which is likely due to the complete diffusion of F^−^ impurities through dynamic “dissolution–recrystallization” of the gypsum phase. The release of PO_4_^3−^ increases rapidly with decreasing pH below 8 and demonstrates high compatibility with the leaching behaviors of metal ions, such as Ca^2+^, Al^3+^, Fe^2+^, and Y^3+^.
(2) The metal leaching behavior in PG substantially affects the release of P and F impurities. Under acidic conditions (pH < 5), SI values for potential fluorine precipitates, such as CaF_2_ and YF_3_, remain relatively low, indicating high fluorine mobility. When the pH exceeds 5, the precipitation rates of CaF_2_ and YF_3_ become the controlling factors for fluorine release. Below pH = 3, phosphate ions react with rare earth metal ions (Y^3+^) to form phosphate precipitates. As the pH increases, calcium phosphates, such as Ca_2_(PO_4_)_3_, are precipitated as the primary phosphorus control species in PG.
(3) FTIR analysis of the “dissolution–recrystallization” of gypsum crystals in PG during static leaching, with a blue shift of the antisymmetric stretching vibration of SO_4_^2−^ from 1142 cm^−1^ to 1078 cm^−1^, indicated a change in the crystal structure. The surface element mapping demonstrates a substantial decrease in the F content on the gypsum crystal surfaces in the pH range of 3–12, highlighting the strong migration potential of fluorine. Additionally, the XPS analysis reveals the formation of AlF_3_ and CaHPO_4_ precipitates on the sample surface under acidic leaching conditions, which precipitates Ca_3_(PO_4_)_2_ at pH = 12.

## Data Availability

The data used in this study are available upon reasonable request from the corresponding author.
